# Supplementary material for: The SAFE Labs Handbook as a tool for improving lab culture
Source: eLife. 2025 Nov 28;14:e108853. doi: 10.7554/eLife.108853 (PMC12662629; doi:10.7554/eLife.108853)
Supplement: Supplementary file 2. [file elife-108853-supp2.pdf]

**Did SAFE Labs meet your overall expectations?**		
**1 = Below to 5 = Exceeded**	5	4
**How much would you agree with the following statements**		
**1 = Strongly Disagree to 5 = Strongly Agree**		
SAFE Labs was a unique meeting that fulfilled an important niche	5	5
Discussions were productive and participants were respectful of each other's opinions	5	4
I learnt something new that I intend to implement in my own lab	5	4
I will change a practice in my lab as a result of attending SAFE Labs	4	4
I will share the outcomes with colleagues in my institution	4	4
I think future iterations of SAFE Labs would be useful for other new PIs	5	4
**Please indicate how useful you found each of the sessions/topics**		
**1 = Useless to 5 = Extremely Valuable**		
Team Management	3	3
Encouraging Feedback	5	4
Career Development	4	5
Recruitment	5	5
Equity, Diversity, Inclusion	4	4
Sustainability & Work-life Balance	4	5
Case Studies	5	4

Responses						
5	5	5	5	5	4	5
5	5	5	4	5	5	5
5	5	5	5	5	3	5
5	5	5	4	5	4	5
5	5	4	4	5	4	5
4	4	5	5	5	4	5
5	5	5	5	5	4	5
4	5	5	5	5	4	5
5	4	5	3	5	5	5
4	5	4	5	5	5	5
4	4	4	4	5	5	5
5	4	5	5	5	4	5
5	5	4	3	5	3	5
5	4	3	5	5	3	5
